# Crystal structure of anagyrine perchlorate

**DOI:** 10.1107/S2056989015007781

**Published:** 2015-04-25

**Authors:** Kambarali K. Turgunov, Shukhrat B. Rakhimov, Valentina I. Vinogradova, Bakhodir Tashkhodjaev

**Affiliations:** a S. Yunusov Institute of the Chemistry of Plant Substances, Academy of Sciences of Uzbekistan, Mirzo Ulugbek Str. 77, Tashkent 100170, Uzbekistan

**Keywords:** crystal structure, alkaloid, *Genista Hispanica*, anagyrine, perchlorate, N—H⋯O hydrogen bonds, π–π stacking inter­actions

## Abstract

The title mol­ecular salt, C_15_H_21_N_2_O^+^·ClO_4_^−^, crystallizes with four cations (*A*, *B*, *C* and *D*) and four anions in the chiral unit cell (space group *P*2_1_). The alkaloid was isolated from the aerial parts of *Genista Hispanica* collected in the Samarkand region of Uzbekistan. Each cation is protonated at the N atom that bridges the alkaloid rings *C* and *D*. In each cation, ring *A* is almost planar and ring *B* adops a sofa conformation with the methyl­ene group bridging to the *C* ring as the flap. Rings *C* and *D* adopt chair conformations with a *cis* ring junction in all four cations. In the crystal, *A*+*B* and *C*+*D* dimeric pairs linked by pairs of N—H⋯O hydrogen bonds are observed, which generate *R*_2_^2^(16) loops in each case. The dimers are consolidated by weak aromatic π–π stacking inter­actions between the *A* rings [centroid–centroid distances = 3.913 (3) and 3.915 (3) Å].

## Related literature   

For the isolation of the title alkaloid, see: Orechoff *et al.* (1934 ▸); Sagen *et al.* (2002 ▸). For NMR spectra of the title alkaloid, see: Sagen *et al.* (2002 ▸). For theoretical studies of anagyrine and the crystal structure of anagyrine hydro­chloride monohydrate, see: Galasso *et al.* (2006 ▸). For a related crystal structure, see: Atta-ur-Rahman *et al.* (1991 ▸).
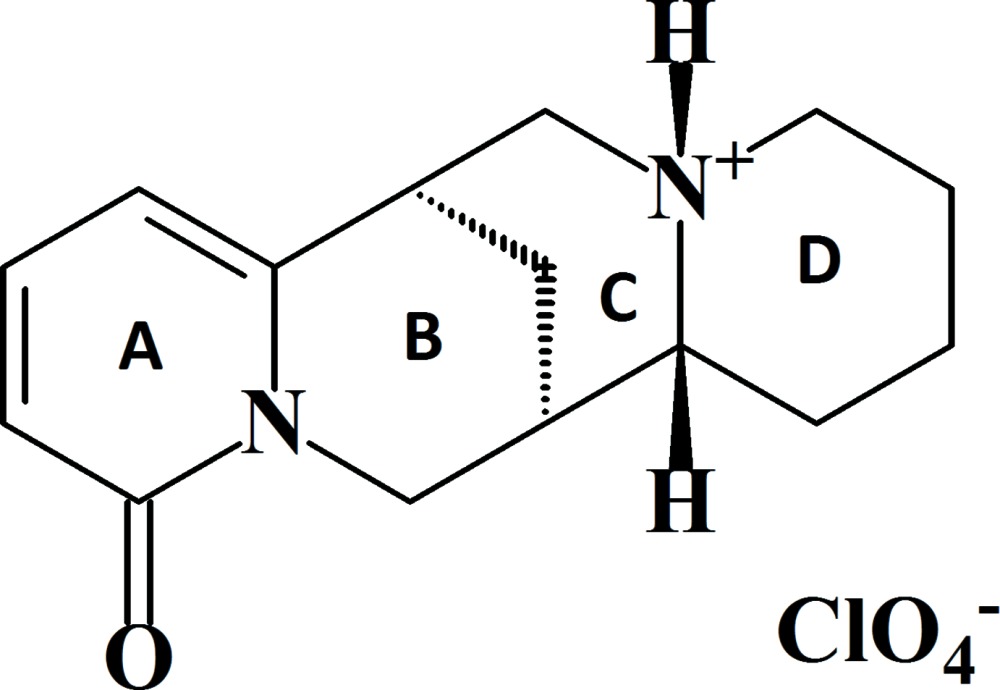


## Experimental   

### Crystal data  

C_15_H_21_N_2_O^+^·ClO_4_^−^
*M* *_r_* = 344.79
Monoclinic, 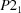

*a* = 7.3550 (3) Å
*b* = 32.982 (1) Å
*c* = 12.8849 (4) Å
β = 90.709 (3)°
*V* = 3125.41 (19) Å^3^
*Z* = 8
Cu *K*α radiation
μ = 2.42 mm^−1^
*T* = 290 K
0.65 × 0.15 × 0.04 mm

### Data collection  

Oxford Diffraction Xcalibur Ruby diffractometer
Absorption correction: multi-scan (*CrysAlis PRO*; Oxford Diffraction, 2009 ▸) *T*_min_ = 0.651, *T*_max_ = 1.000
53652 measured reflections
12780 independent reflections
9677 reflections with *I* > 2σ(*I*)
*R*_int_ = 0.105

### Refinement  

*R*[*F* ^2^ > 2σ(*F* ^2^)] = 0.064
*wR*(*F* ^2^) = 0.189
*S* = 1.02
12780 reflections
846 parameters
1 restraint
H atoms treated by a mixture of independent and constrained refinement
Δρ_max_ = 0.60 e Å^−3^
Δρ_min_ = −0.37 e Å^−3^
Absolute structure: Flack *x* determined using 3107 quotients [(*I* ^+^)−(*I* ^−^)]/[(*I* ^+^)+(*I* ^−^)] (Parsons *et al.*, 2013 ▸)
Absolute structure parameter: −0.024 (12)

### 

Data collection: *CrysAlis PRO* (Oxford Diffraction, 2009 ▸); cell refinement: *CrysAlis PRO*; data reduction: *CrysAlis PRO*; program(s) used to solve structure: *SHELXS97* (Sheldrick, 2008 ▸); program(s) used to refine structure: *SHELXL97* (Sheldrick, 2008 ▸); molecular graphics: *XP* in *SHELXTL* (Sheldrick, 2008 ▸); software used to prepare material for publication: *publCIF* (Westrip, 2010 ▸).

## Supplementary Material

Crystal structure: contains datablock(s) I, New_Global_Publ_Block. DOI: 10.1107/S2056989015007781/hb7409sup1.cif

Structure factors: contains datablock(s) I. DOI: 10.1107/S2056989015007781/hb7409Isup2.hkl

**Fig. 1.**
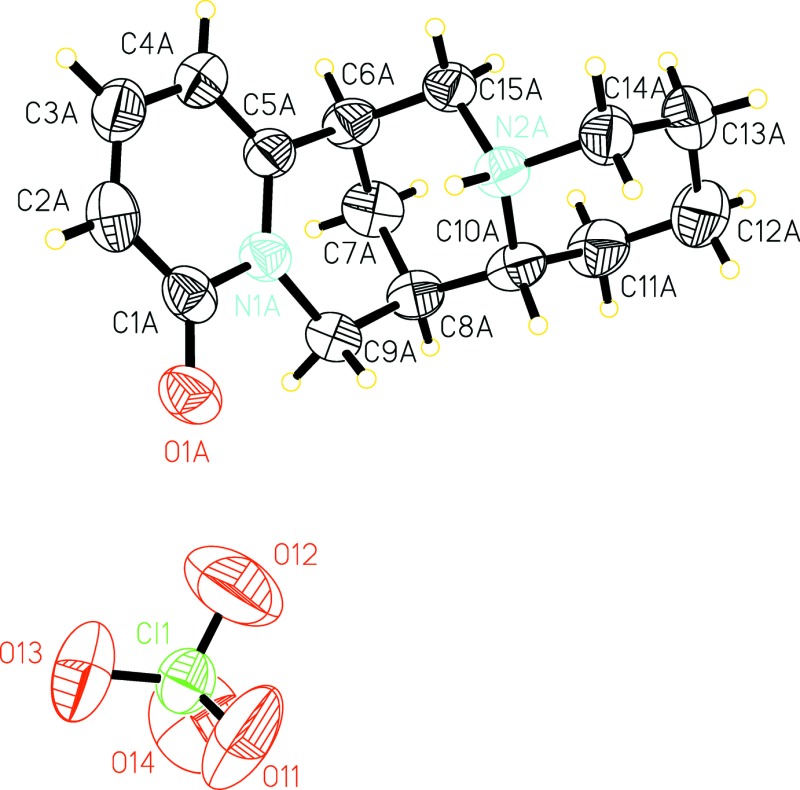
The molecular structure of cation A of the title compound, with displacement ellipsoids drawn at the 50% probability level.

. DOI: 10.1107/S2056989015007781/hb7409fig1.tif
The mol­ecular structure of cation A of the title compound, with displacement ellipsoids drawn at the 50% probability level.

**Fig. 2.**
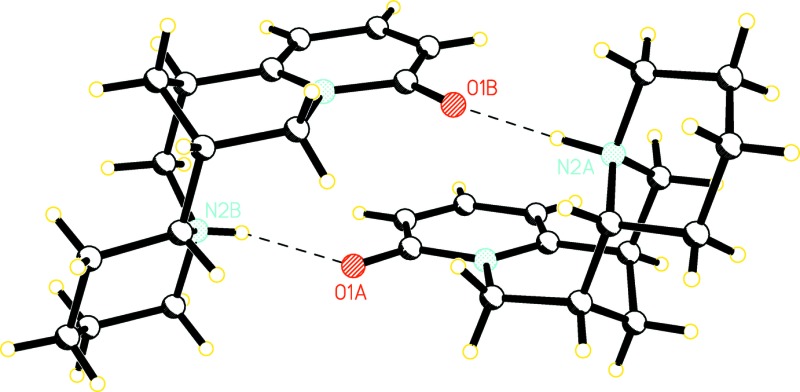
Hydrogen bonding between molecules.

. DOI: 10.1107/S2056989015007781/hb7409fig2.tif
Hydrogen bonding between mol­ecules.

CCDC reference: 1060546

Additional supporting information:  crystallographic information; 3D view; checkCIF report

## Figures and Tables

**Table 1:** Hydrogen-bond geometry (, )

*D*H*A*	*D*H	H*A*	*D* *A*	*D*H*A*
N2*A*H2*AN*O1*B*	1.03(5)	1.91(6)	2.741(6)	136(5)
N2*B*H2*CN*O1*A*	0.77(7)	2.00(6)	2.742(5)	163(6)
N2*C*H2*EN*O1*D* ^i^	0.90(9)	2.00(9)	2.735(6)	138(8)
N2*D*H2*GN*O1*C* ^ii^	1.05(5)	1.74(5)	2.754(5)	159(5)

Symmetry codes: (i) 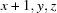; (ii) 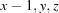.

## supplementary crystallographic information

### S1. Comment

Quinolizidine alkaloids attracted the attention of researchers due to the structural characteristics and pharmacological activity. We have studied the aerial parts of *Genista Hispanica* collected in Samarkand region and isolated anagyrine with R_f_ 0.5 (chloro­form-methanol 6:1) along with other alkaloids. Anagyrine a toxic alkaloid found in several species of *Lupinus* in the western United States. Acute poisoning produces nervousness, depression, loss of muscular control, convulsions, and coma.

Anagyrine perchlorate crystallizes in the monoclinic space group P2_1_ with a long unique b-axis of 32.982 (1) Å .The asymmetric unit consist of four protonated anagyrine molecules and four perchlorate anions. The molecular structure of the alkaloid is shown in Fig. 1. The alkaloid molecule consists of four fused rings - planar ring A fused with the sofa ring B, and a twin-chair C/D fragment where C/D junction is *cis*. Conformation of all undependent molecules matches. Anagyrine molecule has three asymmetric centers at C6, C8, C10 and in addition by protonation of N2 it becomes as asymmetric senter. Configuration of chiral atoms are C-6R,C-8R,C-10R and N-2S. Crystal structure of thermopsine - a C-10-epimer of anagyrine was investigated by Atta-ur-Rahman *et al.* (1991).

In the crystal, pairs of hydrogen bonds between protonated N atom of the base and the carbonyl O atom link molecules to form two molecular associates (Fig.2, Table 1.). In addition the associates are linked by weak π–π stacking inter­actions observed between aromatic rings of molecule [centroid—centroid distance = 3.913 (3) Å and 3.915 (3) Å for undependent molecular pairs ]

### S2.1. Synthesis and crystallization

The powdered air-dried plant material were extracted with 80% ethanol. After distilling off the alcohol, the residue was acidified with H_2_SO_4_ and washed with chloro­form, then the extract was basified with 25% aqueous ammonia, the sum of alkaloids (8.81 g) were extracted with chloro­form. The resulting sum were dissolved in ethanol and acidified with HNO_3_ to a weakly acidic medium, precipitated cytisine nitrate crystals (0.98g) were separated, and the mother liquor was evaporated. The resulting aqueous residue was basified with 25% aqueous ammonia and alkaloids was extracted with chloro­form. The resulting sum of alkaloids was subjected to column chromatography on silica gel eluting with chloro­form-methanol (100: 1) and isolated anagyrine (0.16g). Obtained anagyrine was dissolved in acetone and perchloric acid was added until acidic medium of pH 5-6. Precipitated anagyrine perchlorate crystals were crystallized from methanol with m.p. 315 °C. Colourless prisms were obtained by re-crystallization from water at 50 °C .

### S2.2. Refinement

Carbon-bound H atoms were placed geometrically and treated as riding on their parent atoms, with C—H distances of 0.93 Å (aromatic), 0.97 Å (methylen), 0.98 Å (tertiary carbon) and were refined with Uiso(H)=1.2Ueq(C) for all hydrogen atoms. N-bound H atoms involved in the inter­molecular hydrogen bonding were found by difference Fourier synthesis and refined isotropically [N2A—H = 1.03 (5)Å, N2B—H 0.77 (7) Å, N2C—H 0.90 (9) Å, N2D—H 1.05 (5) Å].

### Crystal data

**Table:**

C_15_H_21_N_2_O^+^·ClO_4_^−^	*F*(000) = 1456
*M**_r_* = 344.79	*D*_x_ = 1.465 Mg m^−^^3^
Monoclinic, *P*2_1_	Melting point: 588(2) K
Hall symbol: P 2yb	Cu *K*α radiation, λ = 1.54184 Å
*a* = 7.3550 (3) Å	Cell parameters from 6414 reflections
*b* = 32.982 (1) Å	θ = 3.7–75.0°
*c* = 12.8849 (4) Å	µ = 2.42 mm^−^^1^
β = 90.709 (3)°	*T* = 290 K
*V* = 3125.41 (19) Å^3^	Prism, colourless
*Z* = 8	0.65 × 0.15 × 0.04 mm

### Data collection

**Table:**

Oxford Diffraction Xcalibur Ruby diffractometer	12780 independent reflections
Radiation source: Enhance (Cu) X-ray Source	9677 reflections with *I* > 2σ(*I*)
Graphite monochromator	*R*_int_ = 0.105
Detector resolution: 10.2576 pixels mm^-1^	θ_max_ = 77.5°, θ_min_ = 3.4°
ω scans	*h* = −9→8
Absorption correction: multi-scan (*CrysAlis PRO*; Oxford Diffraction, 2009)	*k* = −41→41
*T*_min_ = 0.651, *T*_max_ = 1.000	*l* = −16→16
53652 measured reflections	

### Refinement

**Table:**

Refinement on *F*^2^	Hydrogen site location: inferred from neighbouring sites
Least-squares matrix: full	H atoms treated by a mixture of independent and constrained refinement
*R*[*F*^2^ > 2σ(*F*^2^)] = 0.064	*w* = 1/[σ^2^(*F*_o_^2^) + (0.0663*P*)^2^ + 0.7864*P*] where *P* = (*F*_o_^2^ + 2*F*_c_^2^)/3
*wR*(*F*^2^) = 0.189	(Δ/σ)_max_ = 0.005
*S* = 1.02	Δρ_max_ = 0.60 e Å^−^^3^
12780 reflections	Δρ_min_ = −0.37 e Å^−^^3^
846 parameters	Extinction correction: *SHELXL*, Fc^*^=kFc[1+0.001xFc^2^λ^3^/sin(2θ)]^-1/4^
1 restraint	Extinction coefficient: 0.00064 (10)
Primary atom site location: structure-invariant direct methods	Absolute structure: Flack *x* determined using 3107 quotients [(*I*^+^)-(*I*^-^)]/[(*I*^+^)+(*I*^-^)] (Parsons *et al.*, 2013)
Secondary atom site location: difference Fourier map	Absolute structure parameter: −0.024 (12)

### Special details

**Table:**

Geometry. All e.s.d.'s (except the e.s.d. in the dihedral angle between two l.s. planes) are estimated using the full covariance matrix. The cell e.s.d.'s are taken into account individually in the estimation of e.s.d.'s in distances, angles and torsion angles; correlations between e.s.d.'s in cell parameters are only used when they are defined by crystal symmetry. An approximate (isotropic) treatment of cell e.s.d.'s is used for estimating e.s.d.'s involving l.s. planes.
Refinement. Refinement of *F*^2^ against ALL reflections. The weighted *R*-factor *wR* and goodness of fit *S* are based on *F*^2^, conventional *R*-factors *R* are based on *F*, with *F* set to zero for negative *F*^2^. The threshold expression of *F*^2^ > σ(*F*^2^) is used only for calculating *R*-factors(gt) *etc*. and is not relevant to the choice of reflections for refinement. *R*-factors based on *F*^2^ are statistically about twice as large as those based on *F*, and *R*- factors based on ALL data will be even larger.

### Fractional atomic coordinates and isotropic or equivalent isotropic displacement parameters (Å^2^)

**Table:**

	*x*	*y*	*z*	*U*_iso_*/*U*_eq_	
O1A	0.4133 (6)	0.31408 (11)	0.7883 (3)	0.0586 (9)	
N1A	0.5187 (5)	0.35415 (11)	0.9204 (3)	0.0447 (8)	
N2A	0.2907 (6)	0.38902 (11)	1.1098 (3)	0.0460 (8)	
C1A	0.4817 (7)	0.34744 (15)	0.8144 (4)	0.0485 (10)	
C2A	0.5292 (7)	0.37879 (17)	0.7450 (4)	0.0576 (12)	
H2A	0.5092	0.3752	0.6741	0.069*	
C3A	0.6032 (8)	0.41377 (17)	0.7797 (4)	0.0593 (12)	
H3A	0.6344	0.4340	0.7328	0.071*	
C4A	0.6333 (8)	0.41978 (15)	0.8873 (4)	0.0569 (12)	
H4A	0.6803	0.4443	0.9111	0.068*	
C5A	0.5937 (6)	0.38989 (14)	0.9558 (4)	0.0468 (9)	
C6A	0.6195 (7)	0.39559 (14)	1.0709 (4)	0.0509 (10)	
H6A	0.7237	0.4136	1.0823	0.061*	
C7A	0.6605 (8)	0.35544 (16)	1.1242 (4)	0.0585 (12)	
H7A	0.6815	0.3595	1.1979	0.070*	
H7B	0.7678	0.3430	1.0948	0.070*	
C8A	0.4945 (7)	0.32869 (14)	1.1059 (4)	0.0495 (10)	
H8A	0.5179	0.3029	1.1412	0.059*	
C9A	0.4759 (7)	0.31962 (13)	0.9901 (4)	0.0500 (10)	
H9A	0.5558	0.2972	0.9735	0.060*	
H9B	0.3522	0.3109	0.9757	0.060*	
C10A	0.3225 (8)	0.34697 (14)	1.1534 (4)	0.0525 (11)	
H10A	0.2194	0.3300	1.1316	0.063*	
C11A	0.3270 (10)	0.34759 (18)	1.2714 (4)	0.0660 (14)	
H11A	0.4344	0.3621	1.2953	0.079*	
H11B	0.3345	0.3200	1.2973	0.079*	
C12A	0.1551 (12)	0.3683 (2)	1.3153 (5)	0.0811 (19)	
H12A	0.0493	0.3519	1.2989	0.097*	
H12B	0.1663	0.3701	1.3903	0.097*	
C13A	0.1284 (10)	0.41028 (19)	1.2713 (4)	0.0677 (14)	
H13A	0.0151	0.4215	1.2962	0.081*	
H13B	0.2268	0.4277	1.2946	0.081*	
C14A	0.1239 (7)	0.40890 (16)	1.1549 (4)	0.0548 (11)	
H14A	0.1141	0.4363	1.1283	0.066*	
H14B	0.0167	0.3941	1.1321	0.066*	
C15A	0.4529 (7)	0.41532 (14)	1.1185 (3)	0.0479 (10)	
H15A	0.4777	0.4211	1.1911	0.057*	
H15B	0.4285	0.4409	1.0836	0.057*	
O1B	0.0848 (6)	0.36310 (12)	0.9439 (3)	0.0591 (9)	
N1B	0.0180 (6)	0.34775 (12)	0.7754 (3)	0.0464 (8)	
N2B	0.2143 (5)	0.28075 (11)	0.6285 (3)	0.0428 (8)	
C1B	0.0737 (7)	0.37526 (15)	0.8519 (4)	0.0498 (10)	
C2B	0.1114 (8)	0.41506 (17)	0.8180 (5)	0.0633 (13)	
H2C	0.1467	0.4345	0.8664	0.076*	
C3B	0.0975 (9)	0.42544 (18)	0.7174 (5)	0.0682 (14)	
H3C	0.1227	0.4519	0.6974	0.082*	
C4B	0.0450 (9)	0.39665 (17)	0.6415 (5)	0.0648 (14)	
H4C	0.0387	0.4038	0.5717	0.078*	
C5B	0.0042 (8)	0.35841 (16)	0.6721 (4)	0.0538 (11)	
C6B	−0.0512 (8)	0.32671 (17)	0.5943 (4)	0.0559 (12)	
H6C	−0.1171	0.3400	0.5372	0.067*	
C7B	−0.1758 (8)	0.2952 (2)	0.6424 (4)	0.0640 (14)	
H7C	−0.2846	0.3081	0.6686	0.077*	
H7D	−0.2117	0.2752	0.5910	0.077*	
C8B	−0.0701 (7)	0.27531 (16)	0.7304 (4)	0.0546 (11)	
H8C	−0.1510	0.2552	0.7614	0.066*	
C9B	−0.0252 (7)	0.30642 (15)	0.8148 (4)	0.0501 (10)	
H9C	−0.1278	0.3084	0.8611	0.060*	
H9D	0.0780	0.2966	0.8552	0.060*	
C10B	0.0950 (7)	0.25221 (14)	0.6903 (4)	0.0495 (10)	
H10C	0.1657	0.2427	0.7505	0.059*	
C11B	0.0438 (9)	0.21524 (18)	0.6244 (5)	0.0679 (15)	
H11C	−0.0204	0.1959	0.6673	0.082*	
H11D	−0.0384	0.2237	0.5691	0.082*	
C12B	0.2083 (10)	0.19436 (17)	0.5769 (5)	0.0711 (16)	
H12C	0.2831	0.1827	0.6318	0.085*	
H12D	0.1673	0.1725	0.5321	0.085*	
C13B	0.3193 (9)	0.22340 (17)	0.5158 (5)	0.0653 (14)	
H13C	0.2485	0.2332	0.4570	0.078*	
H13D	0.4260	0.2097	0.4896	0.078*	
C14B	0.3771 (7)	0.25872 (16)	0.5831 (4)	0.0561 (11)	
H14C	0.4473	0.2776	0.5420	0.067*	
H14D	0.4546	0.2489	0.6391	0.067*	
C15B	0.1168 (8)	0.30531 (15)	0.5495 (3)	0.0543 (11)	
H15C	0.0787	0.2879	0.4927	0.065*	
H15D	0.1991	0.3255	0.5221	0.065*	
O1C	0.8812 (5)	0.12630 (11)	0.2909 (3)	0.0560 (8)	
N1C	0.7799 (5)	0.08624 (11)	0.4217 (3)	0.0433 (7)	
N2C	1.0207 (6)	0.05052 (11)	0.6095 (3)	0.0456 (8)	
C1C	0.8095 (6)	0.09375 (14)	0.3167 (3)	0.0453 (9)	
C2C	0.7495 (7)	0.06292 (16)	0.2460 (4)	0.0514 (10)	
H2E	0.7659	0.0666	0.1752	0.062*	
C3C	0.6694 (8)	0.02861 (16)	0.2800 (4)	0.0574 (12)	
H3E	0.6292	0.0093	0.2324	0.069*	
C4C	0.6467 (8)	0.02195 (15)	0.3858 (4)	0.0550 (11)	
H4E	0.5940	−0.0020	0.4085	0.066*	
C5C	0.7012 (6)	0.05031 (13)	0.4561 (4)	0.0449 (9)	
C6C	0.6872 (7)	0.04424 (14)	0.5715 (4)	0.0498 (10)	
H6E	0.5835	0.0263	0.5838	0.060*	
C7C	0.6506 (8)	0.08412 (16)	0.6271 (4)	0.0561 (11)	
H7E	0.5412	0.0968	0.5996	0.067*	
H7F	0.6354	0.0795	0.7009	0.067*	
C8C	0.8144 (8)	0.11066 (13)	0.6082 (4)	0.0508 (11)	
H8E	0.7927	0.1364	0.6441	0.061*	
C9C	0.8253 (8)	0.12038 (13)	0.4926 (4)	0.0500 (10)	
H9E	0.7433	0.1427	0.4774	0.060*	
H9F	0.9476	0.1295	0.4778	0.060*	
C10C	0.9917 (7)	0.09288 (14)	0.6542 (4)	0.0509 (10)	
H10E	1.0924	0.1100	0.6312	0.061*	
C11C	0.9978 (10)	0.09176 (17)	0.7727 (4)	0.0647 (14)	
H11E	0.9915	0.1193	0.7989	0.078*	
H11F	0.8920	0.0773	0.7973	0.078*	
C12C	1.1679 (11)	0.0716 (2)	0.8160 (5)	0.0760 (17)	
H12E	1.2733	0.0879	0.7989	0.091*	
H12F	1.1605	0.0701	0.8910	0.091*	
C13C	1.1914 (10)	0.0292 (2)	0.7725 (4)	0.0715 (15)	
H13E	1.0937	0.0119	0.7962	0.086*	
H13F	1.3057	0.0178	0.7970	0.086*	
C14C	1.1893 (8)	0.03078 (18)	0.6545 (4)	0.0598 (12)	
H14E	1.2953	0.0457	0.6316	0.072*	
H14F	1.1983	0.0034	0.6277	0.072*	
C15C	0.8559 (7)	0.02379 (14)	0.6161 (3)	0.0489 (10)	
H15E	0.8351	0.0168	0.6881	0.059*	
H15F	0.8781	−0.0012	0.5785	0.059*	
O1D	0.2197 (6)	0.07898 (11)	0.4471 (3)	0.0569 (8)	
N1D	0.2731 (5)	0.09373 (11)	0.2775 (3)	0.0424 (7)	
N2D	0.0690 (5)	0.16005 (10)	0.1287 (3)	0.0403 (7)	
C1D	0.2222 (7)	0.06657 (14)	0.3553 (3)	0.0464 (10)	
C2D	0.1765 (7)	0.02692 (15)	0.3226 (4)	0.0546 (11)	
H2G	0.1471	0.0074	0.3718	0.065*	
C3D	0.1750 (8)	0.01683 (15)	0.2199 (4)	0.0569 (12)	
H3G	0.1380	−0.0090	0.1996	0.068*	
C4D	0.2279 (8)	0.04458 (15)	0.1455 (4)	0.0540 (11)	
H4G	0.2302	0.0371	0.0759	0.065*	
C5D	0.2762 (7)	0.08259 (14)	0.1743 (3)	0.0461 (9)	
C6D	0.3330 (7)	0.11426 (15)	0.0955 (3)	0.0492 (10)	
H6G	0.3962	0.1004	0.0391	0.059*	
C7D	0.4602 (7)	0.14484 (17)	0.1420 (4)	0.0554 (12)	
H7G	0.4957	0.1644	0.0900	0.066*	
H7H	0.5688	0.1315	0.1685	0.066*	
C8D	0.3609 (7)	0.16602 (14)	0.2301 (4)	0.0508 (10)	
H8G	0.4437	0.1863	0.2599	0.061*	
C9D	0.3199 (7)	0.13482 (14)	0.3155 (3)	0.0480 (10)	
H9G	0.4254	0.1328	0.3611	0.058*	
H9H	0.2198	0.1449	0.3565	0.058*	
C10D	0.1933 (7)	0.18822 (13)	0.1895 (3)	0.0474 (10)	
H10G	0.1259	0.1980	0.2496	0.057*	
C11D	0.2425 (9)	0.22534 (15)	0.1232 (5)	0.0623 (14)	
H11G	0.3119	0.2443	0.1656	0.075*	
H11H	0.3192	0.2167	0.0667	0.075*	
C12D	0.0761 (9)	0.24698 (16)	0.0785 (5)	0.0662 (14)	
H12G	0.0047	0.2582	0.1344	0.079*	
H12H	0.1144	0.2692	0.0344	0.079*	
C13D	−0.0384 (9)	0.21742 (18)	0.0160 (5)	0.0664 (14)	
H13G	0.0306	0.2077	−0.0427	0.080*	
H13H	−0.1460	0.2311	−0.0106	0.080*	
C14D	−0.0952 (7)	0.18134 (16)	0.0832 (4)	0.0540 (11)	
H14G	−0.1719	0.1908	0.1389	0.065*	
H14H	−0.1652	0.1624	0.0414	0.065*	
C15D	0.1650 (7)	0.13525 (14)	0.0495 (3)	0.0498 (10)	
H15G	0.2014	0.1526	−0.0073	0.060*	
H15H	0.0822	0.1149	0.0218	0.060*	
Cl1	0.5283 (2)	0.18310 (5)	0.83543 (11)	0.0683 (3)	
O11	0.3429 (11)	0.1756 (3)	0.8283 (6)	0.150 (4)	
O12	0.5416 (16)	0.2170 (3)	0.8995 (9)	0.181 (5)	
O13	0.6026 (10)	0.1909 (2)	0.7395 (5)	0.120 (2)	
O14	0.6176 (15)	0.1521 (3)	0.8875 (7)	0.161 (4)	
Cl2	0.6107 (3)	0.42676 (4)	0.45172 (10)	0.0705 (4)	
O21	0.452 (2)	0.4201 (8)	0.4894 (10)	0.355 (15)	
O22	0.590 (2)	0.4423 (4)	0.3594 (7)	0.244 (8)	
O23	0.670 (2)	0.3903 (3)	0.4433 (15)	0.298 (11)	
O24	0.7186 (14)	0.4502 (2)	0.5180 (6)	0.153 (4)	
Cl3	0.7597 (2)	0.25649 (4)	0.33650 (10)	0.0654 (3)	
O31	0.6728 (15)	0.2873 (3)	0.3883 (7)	0.167 (4)	
O32	0.7512 (19)	0.2224 (3)	0.3989 (9)	0.195 (5)	
O33	0.6803 (8)	0.24878 (19)	0.2401 (4)	0.1007 (18)	
O34	0.9439 (11)	0.2649 (4)	0.3267 (6)	0.182 (5)	
Cl4	0.7284 (3)	0.02077 (4)	0.96609 (10)	0.0700 (4)	
O41	0.8759 (18)	−0.0026 (3)	1.0007 (9)	0.196 (6)	
O42	0.5883 (17)	0.0062 (4)	1.0232 (7)	0.198 (6)	
O43	0.7115 (10)	0.01230 (17)	0.8606 (3)	0.0973 (18)	
O44	0.7781 (14)	0.06101 (17)	0.9878 (4)	0.135 (3)	
H2AN	0.259 (8)	0.3892 (17)	1.032 (4)	0.051 (14)*	
H2CN	0.264 (9)	0.2945 (18)	0.668 (5)	0.052 (15)*	
H2EN	1.034 (13)	0.055 (3)	0.541 (7)	0.11 (3)*	
H2GN	0.015 (7)	0.1414 (16)	0.187 (4)	0.043 (13)*	

### Atomic displacement parameters (Å^2^)

**Table:**

	*U*^11^	*U*^22^	*U*^33^	*U*^12^	*U*^13^	*U*^23^
O1A	0.060 (2)	0.0617 (19)	0.0538 (19)	−0.0031 (16)	−0.0063 (16)	−0.0159 (15)
N1A	0.040 (2)	0.0453 (17)	0.0491 (19)	−0.0026 (15)	0.0007 (15)	−0.0076 (14)
N2A	0.050 (2)	0.0452 (17)	0.0423 (18)	−0.0046 (16)	−0.0050 (16)	0.0012 (14)
C1A	0.038 (2)	0.055 (2)	0.053 (2)	0.0049 (19)	−0.0001 (18)	−0.0126 (19)
C2A	0.048 (3)	0.074 (3)	0.051 (3)	0.010 (2)	0.007 (2)	−0.002 (2)
C3A	0.058 (3)	0.062 (3)	0.059 (3)	0.001 (2)	0.015 (2)	0.006 (2)
C4A	0.058 (3)	0.055 (3)	0.058 (3)	−0.010 (2)	0.013 (2)	−0.002 (2)
C5A	0.040 (2)	0.045 (2)	0.055 (2)	−0.0056 (18)	0.0040 (18)	−0.0063 (18)
C6A	0.045 (3)	0.052 (2)	0.056 (3)	−0.0108 (19)	−0.0066 (19)	−0.0060 (19)
C7A	0.053 (3)	0.062 (3)	0.061 (3)	0.003 (2)	−0.017 (2)	−0.001 (2)
C8A	0.051 (3)	0.045 (2)	0.053 (2)	−0.0014 (18)	−0.008 (2)	0.0045 (17)
C9A	0.054 (3)	0.044 (2)	0.052 (2)	0.0011 (19)	−0.002 (2)	−0.0052 (17)
C10A	0.061 (3)	0.046 (2)	0.051 (2)	−0.014 (2)	−0.008 (2)	0.0050 (18)
C11A	0.090 (4)	0.062 (3)	0.045 (3)	−0.007 (3)	−0.003 (3)	0.014 (2)
C12A	0.107 (6)	0.081 (4)	0.055 (3)	−0.011 (4)	0.023 (3)	0.011 (3)
C13A	0.076 (4)	0.071 (3)	0.057 (3)	0.003 (3)	0.016 (3)	−0.007 (2)
C14A	0.054 (3)	0.059 (3)	0.051 (2)	−0.003 (2)	0.006 (2)	−0.001 (2)
C15A	0.051 (3)	0.047 (2)	0.045 (2)	−0.0101 (19)	−0.0018 (18)	−0.0063 (16)
O1B	0.061 (2)	0.070 (2)	0.0461 (18)	0.0027 (17)	−0.0119 (15)	−0.0126 (15)
N1B	0.044 (2)	0.0549 (19)	0.0403 (18)	0.0049 (16)	−0.0033 (15)	−0.0072 (15)
N2B	0.045 (2)	0.0442 (17)	0.0396 (17)	−0.0071 (15)	−0.0041 (15)	−0.0049 (14)
C1B	0.039 (2)	0.060 (2)	0.050 (2)	0.0102 (19)	−0.0046 (18)	−0.0109 (19)
C2B	0.053 (3)	0.055 (3)	0.081 (4)	0.003 (2)	−0.001 (3)	−0.018 (2)
C3B	0.060 (4)	0.056 (3)	0.088 (4)	0.005 (2)	0.011 (3)	0.009 (3)
C4B	0.072 (4)	0.064 (3)	0.058 (3)	0.020 (3)	0.001 (3)	0.007 (2)
C5B	0.056 (3)	0.063 (3)	0.042 (2)	0.011 (2)	−0.002 (2)	0.0016 (19)
C6B	0.056 (3)	0.071 (3)	0.041 (2)	0.013 (2)	−0.013 (2)	−0.005 (2)
C7B	0.039 (3)	0.090 (4)	0.063 (3)	−0.001 (2)	−0.001 (2)	−0.027 (3)
C8B	0.052 (3)	0.062 (3)	0.051 (2)	−0.018 (2)	0.007 (2)	−0.010 (2)
C9B	0.050 (3)	0.060 (3)	0.040 (2)	−0.006 (2)	0.0037 (18)	−0.0037 (18)
C10B	0.053 (3)	0.047 (2)	0.049 (2)	−0.0091 (19)	0.0043 (19)	−0.0035 (17)
C11B	0.067 (4)	0.062 (3)	0.076 (4)	−0.025 (3)	0.008 (3)	−0.015 (3)
C12B	0.083 (4)	0.055 (3)	0.075 (4)	−0.005 (3)	−0.004 (3)	−0.026 (3)
C13B	0.061 (3)	0.062 (3)	0.073 (3)	−0.001 (2)	0.003 (3)	−0.025 (3)
C14B	0.050 (3)	0.057 (3)	0.062 (3)	0.006 (2)	−0.003 (2)	−0.013 (2)
C15B	0.067 (3)	0.061 (3)	0.035 (2)	0.001 (2)	0.002 (2)	0.0004 (18)
O1C	0.057 (2)	0.0607 (19)	0.0500 (17)	−0.0013 (16)	0.0074 (15)	0.0148 (14)
N1C	0.043 (2)	0.0449 (17)	0.0420 (18)	−0.0007 (15)	0.0033 (14)	0.0020 (14)
N2C	0.053 (2)	0.0494 (19)	0.0346 (17)	−0.0036 (16)	0.0057 (15)	0.0040 (14)
C1C	0.041 (2)	0.054 (2)	0.041 (2)	0.0054 (19)	−0.0002 (17)	0.0071 (17)
C2C	0.046 (3)	0.067 (3)	0.041 (2)	0.007 (2)	−0.0032 (18)	−0.0056 (19)
C3C	0.056 (3)	0.059 (3)	0.056 (3)	0.009 (2)	−0.010 (2)	−0.014 (2)
C4C	0.057 (3)	0.048 (2)	0.061 (3)	−0.007 (2)	0.000 (2)	−0.007 (2)
C5C	0.036 (2)	0.050 (2)	0.049 (2)	−0.0040 (17)	0.0008 (17)	0.0022 (17)
C6C	0.051 (3)	0.048 (2)	0.050 (2)	−0.0107 (19)	0.013 (2)	0.0030 (18)
C7C	0.054 (3)	0.063 (3)	0.052 (3)	−0.004 (2)	0.014 (2)	0.001 (2)
C8C	0.061 (3)	0.042 (2)	0.050 (2)	−0.0019 (19)	0.011 (2)	−0.0077 (17)
C9C	0.059 (3)	0.045 (2)	0.047 (2)	−0.0036 (19)	−0.001 (2)	0.0022 (17)
C10C	0.060 (3)	0.050 (2)	0.043 (2)	−0.011 (2)	0.0060 (19)	0.0022 (17)
C11C	0.092 (4)	0.061 (3)	0.042 (2)	−0.005 (3)	0.003 (2)	−0.007 (2)
C12C	0.089 (5)	0.088 (4)	0.050 (3)	−0.013 (4)	−0.013 (3)	−0.002 (3)
C13C	0.077 (4)	0.087 (4)	0.050 (3)	0.004 (3)	−0.004 (3)	0.011 (3)
C14C	0.057 (3)	0.068 (3)	0.055 (3)	0.009 (2)	0.004 (2)	0.011 (2)
C15C	0.057 (3)	0.046 (2)	0.044 (2)	−0.004 (2)	0.0043 (19)	0.0037 (17)
O1D	0.066 (2)	0.0650 (19)	0.0397 (16)	0.0003 (17)	0.0116 (15)	0.0094 (14)
N1D	0.042 (2)	0.0485 (17)	0.0369 (16)	0.0005 (15)	0.0040 (14)	0.0026 (13)
N2D	0.0384 (19)	0.0408 (16)	0.0419 (17)	−0.0041 (14)	0.0030 (14)	0.0061 (13)
C1D	0.046 (3)	0.053 (2)	0.041 (2)	0.0062 (19)	0.0088 (17)	0.0103 (17)
C2D	0.051 (3)	0.051 (2)	0.061 (3)	0.004 (2)	0.005 (2)	0.011 (2)
C3D	0.062 (3)	0.044 (2)	0.065 (3)	0.006 (2)	0.007 (2)	0.000 (2)
C4D	0.060 (3)	0.056 (2)	0.045 (2)	0.010 (2)	0.003 (2)	−0.0046 (19)
C5D	0.045 (2)	0.055 (2)	0.038 (2)	0.0077 (19)	0.0029 (17)	0.0017 (17)
C6D	0.051 (3)	0.060 (2)	0.0372 (19)	0.002 (2)	0.0140 (18)	0.0031 (17)
C7D	0.031 (2)	0.075 (3)	0.059 (3)	−0.007 (2)	0.0002 (19)	0.028 (2)
C8D	0.047 (3)	0.053 (2)	0.051 (2)	−0.0144 (19)	−0.0042 (19)	0.0082 (19)
C9D	0.049 (3)	0.058 (2)	0.037 (2)	−0.006 (2)	−0.0053 (17)	0.0024 (17)
C10D	0.057 (3)	0.042 (2)	0.043 (2)	−0.0100 (19)	0.0025 (18)	0.0010 (16)
C11D	0.073 (4)	0.049 (2)	0.065 (3)	−0.018 (2)	−0.008 (3)	0.014 (2)
C12D	0.080 (4)	0.047 (2)	0.071 (3)	−0.008 (2)	0.004 (3)	0.016 (2)
C13D	0.067 (4)	0.068 (3)	0.065 (3)	−0.002 (3)	−0.006 (3)	0.025 (3)
C14D	0.047 (3)	0.056 (2)	0.059 (3)	−0.001 (2)	0.002 (2)	0.018 (2)
C15D	0.059 (3)	0.056 (2)	0.0351 (19)	0.004 (2)	0.0044 (18)	0.0027 (17)
Cl1	0.0701 (9)	0.0747 (8)	0.0602 (7)	−0.0005 (7)	0.0010 (6)	0.0107 (6)
O11	0.099 (5)	0.237 (10)	0.114 (5)	−0.075 (6)	0.002 (4)	0.053 (6)
O12	0.197 (11)	0.136 (7)	0.211 (10)	0.006 (7)	0.029 (8)	−0.090 (7)
O13	0.106 (5)	0.149 (6)	0.105 (4)	0.017 (4)	0.039 (4)	0.050 (4)
O14	0.202 (10)	0.155 (7)	0.124 (6)	0.066 (7)	−0.028 (6)	0.041 (5)
Cl2	0.1003 (12)	0.0585 (6)	0.0522 (6)	0.0064 (7)	−0.0139 (7)	−0.0059 (5)
O21	0.232 (16)	0.68 (4)	0.151 (10)	−0.22 (2)	0.081 (10)	−0.093 (17)
O22	0.350 (16)	0.269 (13)	0.110 (6)	−0.210 (13)	−0.120 (8)	0.074 (7)
O23	0.331 (19)	0.117 (7)	0.44 (2)	0.107 (10)	−0.243 (18)	−0.132 (10)
O24	0.243 (10)	0.091 (4)	0.121 (5)	−0.004 (5)	−0.107 (6)	−0.023 (4)
Cl3	0.0663 (8)	0.0713 (7)	0.0584 (7)	0.0034 (6)	−0.0049 (6)	−0.0103 (5)
O31	0.206 (10)	0.174 (8)	0.122 (6)	0.079 (7)	−0.004 (6)	−0.070 (6)
O32	0.274 (15)	0.119 (6)	0.192 (10)	0.028 (8)	−0.049 (9)	0.059 (6)
O33	0.092 (4)	0.119 (4)	0.090 (3)	0.020 (3)	−0.037 (3)	−0.027 (3)
O34	0.088 (5)	0.338 (15)	0.119 (5)	−0.068 (7)	0.002 (4)	−0.092 (7)
Cl4	0.1067 (12)	0.0591 (6)	0.0445 (6)	−0.0147 (7)	0.0163 (6)	−0.0037 (5)
O41	0.260 (14)	0.134 (7)	0.192 (10)	0.046 (8)	−0.121 (10)	−0.031 (7)
O42	0.242 (12)	0.241 (11)	0.113 (5)	−0.130 (10)	0.107 (7)	−0.060 (6)
O43	0.148 (5)	0.097 (3)	0.047 (2)	−0.033 (3)	0.012 (3)	−0.003 (2)
O44	0.268 (11)	0.069 (3)	0.068 (3)	−0.033 (4)	0.011 (4)	−0.012 (2)

### Geometric parameters (Å, º)

**Table:**

O1A—C1A	1.254 (6)	C1C—C2C	1.432 (7)
N1A—C5A	1.377 (6)	C2C—C3C	1.351 (8)
N1A—C1A	1.407 (6)	C2C—H2E	0.9300
N1A—C9A	1.487 (6)	C3C—C4C	1.393 (8)
N2A—C15A	1.478 (6)	C3C—H3E	0.9300
N2A—C14A	1.513 (7)	C4C—C5C	1.359 (7)
N2A—C10A	1.514 (6)	C4C—H4E	0.9300
N2A—H2AN	1.03 (6)	C5C—C6C	1.504 (6)
C1A—C2A	1.414 (8)	C6C—C15C	1.520 (7)
C2A—C3A	1.349 (8)	C6C—C7C	1.524 (7)
C2A—H2A	0.9300	C6C—H6E	0.9800
C3A—C4A	1.414 (8)	C7C—C8C	1.512 (7)
C3A—H3A	0.9300	C7C—H7E	0.9700
C4A—C5A	1.357 (7)	C7C—H7F	0.9700
C4A—H4A	0.9300	C8C—C9C	1.526 (6)
C5A—C6A	1.505 (7)	C8C—C10C	1.541 (8)
C6A—C7A	1.520 (7)	C8C—H8E	0.9800
C6A—C15A	1.523 (7)	C9C—H9E	0.9700
C6A—H6A	0.9800	C9C—H9F	0.9700
C7A—C8A	1.522 (7)	C10C—C11C	1.527 (6)
C7A—H7A	0.9700	C10C—H10E	0.9800
C7A—H7B	0.9700	C11C—C12C	1.518 (10)
C8A—C9A	1.526 (7)	C11C—H11E	0.9700
C8A—C10A	1.536 (8)	C11C—H11F	0.9700
C8A—H8A	0.9800	C12C—C13C	1.517 (10)
C9A—H9A	0.9700	C12C—H12E	0.9700
C9A—H9B	0.9700	C12C—H12F	0.9700
C10A—C11A	1.521 (7)	C13C—C14C	1.521 (7)
C10A—H10A	0.9800	C13C—H13E	0.9700
C11A—C12A	1.550 (10)	C13C—H13F	0.9700
C11A—H11A	0.9700	C14C—H14E	0.9700
C11A—H11B	0.9700	C14C—H14F	0.9700
C12A—C13A	1.507 (9)	C15C—H15E	0.9700
C12A—H12A	0.9700	C15C—H15F	0.9700
C12A—H12B	0.9700	O1D—C1D	1.252 (6)
C13A—C14A	1.500 (7)	N1D—C5D	1.380 (6)
C13A—H13A	0.9700	N1D—C1D	1.399 (5)
C13A—H13B	0.9700	N1D—C9D	1.480 (6)
C14A—H14A	0.9700	N2D—C15D	1.492 (6)
C14A—H14B	0.9700	N2D—C14D	1.509 (6)
C15A—H15A	0.9700	N2D—C10D	1.515 (6)
C15A—H15B	0.9700	N2D—H2GN	1.05 (5)
O1B—C1B	1.253 (6)	C1D—C2D	1.413 (7)
N1B—C5B	1.379 (6)	C2D—C3D	1.365 (8)
N1B—C1B	1.397 (6)	C2D—H2G	0.9300
N1B—C9B	1.490 (6)	C3D—C4D	1.384 (7)
N2B—C15B	1.479 (6)	C3D—H3G	0.9300
N2B—C10B	1.519 (6)	C4D—C5D	1.353 (7)
N2B—C14B	1.524 (6)	C4D—H4G	0.9300
N2B—H2CN	0.77 (6)	C5D—C6D	1.519 (6)
C1B—C2B	1.412 (8)	C6D—C7D	1.496 (7)
C2B—C3B	1.343 (9)	C6D—C15D	1.529 (7)
C2B—H2C	0.9300	C6D—H6G	0.9800
C3B—C4B	1.414 (9)	C7D—C8D	1.526 (8)
C3B—H3C	0.9300	C7D—H7G	0.9700
C4B—C5B	1.356 (8)	C7D—H7H	0.9700
C4B—H4C	0.9300	C8D—C10D	1.521 (7)
C5B—C6B	1.502 (7)	C8D—C9D	1.539 (6)
C6B—C7B	1.522 (9)	C8D—H8G	0.9800
C6B—C15B	1.541 (8)	C9D—H9G	0.9700
C6B—H6C	0.9800	C9D—H9H	0.9700
C7B—C8B	1.516 (8)	C10D—C11D	1.538 (6)
C7B—H7C	0.9700	C10D—H10G	0.9800
C7B—H7D	0.9700	C11D—C12D	1.524 (9)
C8B—C10B	1.528 (8)	C11D—H11G	0.9700
C8B—C9B	1.529 (6)	C11D—H11H	0.9700
C8B—H8C	0.9800	C12D—C13D	1.514 (9)
C9B—H9C	0.9700	C12D—H12G	0.9700
C9B—H9D	0.9700	C12D—H12H	0.9700
C10B—C11B	1.530 (6)	C13D—C14D	1.533 (7)
C10B—H10C	0.9800	C13D—H13G	0.9700
C11B—C12B	1.527 (9)	C13D—H13H	0.9700
C11B—H11C	0.9700	C14D—H14G	0.9700
C11B—H11D	0.9700	C14D—H14H	0.9700
C12B—C13B	1.490 (9)	C15D—H15G	0.9700
C12B—H12C	0.9700	C15D—H15H	0.9700
C12B—H12D	0.9700	Cl1—O13	1.381 (6)
C13B—C14B	1.510 (7)	Cl1—O14	1.385 (7)
C13B—H13C	0.9700	Cl1—O11	1.387 (7)
C13B—H13D	0.9700	Cl1—O12	1.393 (8)
C14B—H14C	0.9700	Cl2—O23	1.285 (8)
C14B—H14D	0.9700	Cl2—O21	1.290 (13)
C15B—H15C	0.9700	Cl2—O22	1.302 (8)
C15B—H15D	0.9700	Cl2—O24	1.392 (6)
O1C—C1C	1.243 (6)	Cl3—O31	1.377 (7)
N1C—C5C	1.394 (6)	Cl3—O32	1.385 (9)
N1C—C1C	1.395 (6)	Cl3—O33	1.389 (5)
N1C—C9C	1.486 (6)	Cl3—O34	1.390 (8)
N2C—C15C	1.502 (6)	Cl4—O42	1.362 (8)
N2C—C14C	1.510 (7)	Cl4—O43	1.391 (5)
N2C—C10C	1.527 (6)	Cl4—O41	1.400 (10)
N2C—H2EN	0.91 (9)	Cl4—O44	1.404 (5)
			
C5A—N1A—C1A	121.9 (4)	C3C—C2C—C1C	121.4 (4)
C5A—N1A—C9A	122.9 (4)	C3C—C2C—H2E	119.3
C1A—N1A—C9A	115.2 (4)	C1C—C2C—H2E	119.3
C15A—N2A—C14A	112.0 (4)	C2C—C3C—C4C	120.5 (5)
C15A—N2A—C10A	112.9 (4)	C2C—C3C—H3E	119.8
C14A—N2A—C10A	112.1 (4)	C4C—C3C—H3E	119.8
C15A—N2A—H2AN	104 (3)	C5C—C4C—C3C	120.4 (5)
C14A—N2A—H2AN	101 (3)	C5C—C4C—H4E	119.8
C10A—N2A—H2AN	114 (3)	C3C—C4C—H4E	119.8
O1A—C1A—N1A	118.1 (5)	C4C—C5C—N1C	119.5 (4)
O1A—C1A—C2A	125.0 (5)	C4C—C5C—C6C	122.9 (4)
N1A—C1A—C2A	116.9 (4)	N1C—C5C—C6C	117.5 (4)
C3A—C2A—C1A	121.1 (5)	C5C—C6C—C15C	111.5 (4)
C3A—C2A—H2A	119.4	C5C—C6C—C7C	111.4 (4)
C1A—C2A—H2A	119.4	C15C—C6C—C7C	110.7 (4)
C2A—C3A—C4A	120.2 (5)	C5C—C6C—H6E	107.7
C2A—C3A—H3A	119.9	C15C—C6C—H6E	107.7
C4A—C3A—H3A	119.9	C7C—C6C—H6E	107.7
C5A—C4A—C3A	120.2 (5)	C8C—C7C—C6C	106.1 (4)
C5A—C4A—H4A	119.9	C8C—C7C—H7E	110.5
C3A—C4A—H4A	119.9	C6C—C7C—H7E	110.5
C4A—C5A—N1A	119.6 (4)	C8C—C7C—H7F	110.5
C4A—C5A—C6A	121.7 (4)	C6C—C7C—H7F	110.5
N1A—C5A—C6A	118.6 (4)	H7E—C7C—H7F	108.7
C5A—C6A—C7A	111.0 (4)	C7C—C8C—C9C	109.3 (4)
C5A—C6A—C15A	111.0 (4)	C7C—C8C—C10C	112.9 (4)
C7A—C6A—C15A	110.3 (4)	C9C—C8C—C10C	113.6 (4)
C5A—C6A—H6A	108.2	C7C—C8C—H8E	106.8
C7A—C6A—H6A	108.2	C9C—C8C—H8E	106.8
C15A—C6A—H6A	108.2	C10C—C8C—H8E	106.8
C6A—C7A—C8A	106.3 (4)	N1C—C9C—C8C	115.3 (4)
C6A—C7A—H7A	110.5	N1C—C9C—H9E	108.5
C8A—C7A—H7A	110.5	C8C—C9C—H9E	108.5
C6A—C7A—H7B	110.5	N1C—C9C—H9F	108.5
C8A—C7A—H7B	110.5	C8C—C9C—H9F	108.5
H7A—C7A—H7B	108.7	H9E—C9C—H9F	107.5
C7A—C8A—C9A	109.1 (4)	N2C—C10C—C11C	110.6 (4)
C7A—C8A—C10A	111.9 (4)	N2C—C10C—C8C	108.9 (4)
C9A—C8A—C10A	113.7 (4)	C11C—C10C—C8C	114.1 (5)
C7A—C8A—H8A	107.3	N2C—C10C—H10E	107.7
C9A—C8A—H8A	107.3	C11C—C10C—H10E	107.7
C10A—C8A—H8A	107.3	C8C—C10C—H10E	107.7
N1A—C9A—C8A	115.0 (4)	C12C—C11C—C10C	113.1 (5)
N1A—C9A—H9A	108.5	C12C—C11C—H11E	109.0
C8A—C9A—H9A	108.5	C10C—C11C—H11E	109.0
N1A—C9A—H9B	108.5	C12C—C11C—H11F	109.0
C8A—C9A—H9B	108.5	C10C—C11C—H11F	109.0
H9A—C9A—H9B	107.5	H11E—C11C—H11F	107.8
N2A—C10A—C11A	111.1 (4)	C13C—C12C—C11C	111.4 (5)
N2A—C10A—C8A	109.6 (4)	C13C—C12C—H12E	109.3
C11A—C10A—C8A	113.4 (5)	C11C—C12C—H12E	109.3
N2A—C10A—H10A	107.5	C13C—C12C—H12F	109.3
C11A—C10A—H10A	107.5	C11C—C12C—H12F	109.3
C8A—C10A—H10A	107.5	H12E—C12C—H12F	108.0
C10A—C11A—C12A	111.3 (5)	C12C—C13C—C14C	109.7 (5)
C10A—C11A—H11A	109.4	C12C—C13C—H13E	109.7
C12A—C11A—H11A	109.4	C14C—C13C—H13E	109.7
C10A—C11A—H11B	109.4	C12C—C13C—H13F	109.7
C12A—C11A—H11B	109.4	C14C—C13C—H13F	109.7
H11A—C11A—H11B	108.0	H13E—C13C—H13F	108.2
C13A—C12A—C11A	111.8 (5)	N2C—C14C—C13C	113.3 (5)
C13A—C12A—H12A	109.3	N2C—C14C—H14E	108.9
C11A—C12A—H12A	109.3	C13C—C14C—H14E	108.9
C13A—C12A—H12B	109.3	N2C—C14C—H14F	108.9
C11A—C12A—H12B	109.3	C13C—C14C—H14F	108.9
H12A—C12A—H12B	107.9	H14E—C14C—H14F	107.7
C14A—C13A—C12A	110.5 (5)	N2C—C15C—C6C	112.0 (4)
C14A—C13A—H13A	109.6	N2C—C15C—H15E	109.2
C12A—C13A—H13A	109.6	C6C—C15C—H15E	109.2
C14A—C13A—H13B	109.6	N2C—C15C—H15F	109.2
C12A—C13A—H13B	109.6	C6C—C15C—H15F	109.2
H13A—C13A—H13B	108.1	H15E—C15C—H15F	107.9
C13A—C14A—N2A	112.9 (5)	C5D—N1D—C1D	121.8 (4)
C13A—C14A—H14A	109.0	C5D—N1D—C9D	123.8 (4)
N2A—C14A—H14A	109.0	C1D—N1D—C9D	114.4 (4)
C13A—C14A—H14B	109.0	C15D—N2D—C14D	111.9 (4)
N2A—C14A—H14B	109.0	C15D—N2D—C10D	113.7 (4)
H14A—C14A—H14B	107.8	C14D—N2D—C10D	113.0 (4)
N2A—C15A—C6A	111.8 (4)	C15D—N2D—H2GN	111 (3)
N2A—C15A—H15A	109.3	C14D—N2D—H2GN	104 (3)
C6A—C15A—H15A	109.3	C10D—N2D—H2GN	103 (3)
N2A—C15A—H15B	109.3	O1D—C1D—N1D	118.4 (4)
C6A—C15A—H15B	109.3	O1D—C1D—C2D	125.3 (4)
H15A—C15A—H15B	107.9	N1D—C1D—C2D	116.4 (4)
C5B—N1B—C1B	122.2 (4)	C3D—C2D—C1D	120.9 (5)
C5B—N1B—C9B	123.3 (4)	C3D—C2D—H2G	119.6
C1B—N1B—C9B	114.6 (4)	C1D—C2D—H2G	119.6
C15B—N2B—C10B	114.9 (4)	C2D—C3D—C4D	120.7 (5)
C15B—N2B—C14B	111.9 (4)	C2D—C3D—H3G	119.6
C10B—N2B—C14B	111.6 (4)	C4D—C3D—H3G	119.6
C15B—N2B—H2CN	111 (4)	C5D—C4D—C3D	119.9 (5)
C10B—N2B—H2CN	107 (4)	C5D—C4D—H4G	120.1
C14B—N2B—H2CN	100 (5)	C3D—C4D—H4G	120.1
O1B—C1B—N1B	118.3 (5)	C4D—C5D—N1D	120.2 (4)
O1B—C1B—C2B	125.4 (5)	C4D—C5D—C6D	121.8 (4)
N1B—C1B—C2B	116.3 (5)	N1D—C5D—C6D	118.0 (4)
C3B—C2B—C1B	121.5 (5)	C7D—C6D—C5D	111.8 (4)
C3B—C2B—H2C	119.3	C7D—C6D—C15D	110.4 (4)
C1B—C2B—H2C	119.3	C5D—C6D—C15D	110.0 (4)
C2B—C3B—C4B	120.9 (6)	C7D—C6D—H6G	108.2
C2B—C3B—H3C	119.6	C5D—C6D—H6G	108.2
C4B—C3B—H3C	119.6	C15D—C6D—H6G	108.2
C5B—C4B—C3B	118.8 (5)	C6D—C7D—C8D	107.7 (4)
C5B—C4B—H4C	120.6	C6D—C7D—H7G	110.2
C3B—C4B—H4C	120.6	C8D—C7D—H7G	110.2
C4B—C5B—N1B	120.3 (5)	C6D—C7D—H7H	110.2
C4B—C5B—C6B	120.8 (5)	C8D—C7D—H7H	110.2
N1B—C5B—C6B	118.9 (5)	H7G—C7D—H7H	108.5
C5B—C6B—C7B	111.2 (4)	C10D—C8D—C7D	111.0 (4)
C5B—C6B—C15B	110.9 (4)	C10D—C8D—C9D	113.8 (4)
C7B—C6B—C15B	109.3 (5)	C7D—C8D—C9D	109.0 (4)
C5B—C6B—H6C	108.5	C10D—C8D—H8G	107.6
C7B—C6B—H6C	108.5	C7D—C8D—H8G	107.6
C15B—C6B—H6C	108.5	C9D—C8D—H8G	107.6
C8B—C7B—C6B	107.1 (4)	N1D—C9D—C8D	115.0 (4)
C8B—C7B—H7C	110.3	N1D—C9D—H9G	108.5
C6B—C7B—H7C	110.3	C8D—C9D—H9G	108.5
C8B—C7B—H7D	110.3	N1D—C9D—H9H	108.5
C6B—C7B—H7D	110.3	C8D—C9D—H9H	108.5
H7C—C7B—H7D	108.5	H9G—C9D—H9H	107.5
C7B—C8B—C10B	111.5 (4)	N2D—C10D—C8D	111.3 (4)
C7B—C8B—C9B	110.2 (4)	N2D—C10D—C11D	110.2 (4)
C10B—C8B—C9B	114.2 (4)	C8D—C10D—C11D	112.3 (4)
C7B—C8B—H8C	106.9	N2D—C10D—H10G	107.6
C10B—C8B—H8C	106.9	C8D—C10D—H10G	107.6
C9B—C8B—H8C	106.9	C11D—C10D—H10G	107.6
N1B—C9B—C8B	114.6 (4)	C12D—C11D—C10D	112.9 (5)
N1B—C9B—H9C	108.6	C12D—C11D—H11G	109.0
C8B—C9B—H9C	108.6	C10D—C11D—H11G	109.0
N1B—C9B—H9D	108.6	C12D—C11D—H11H	109.0
C8B—C9B—H9D	108.6	C10D—C11D—H11H	109.0
H9C—C9B—H9D	107.6	H11G—C11D—H11H	107.8
N2B—C10B—C8B	109.5 (4)	C13D—C12D—C11D	109.8 (5)
N2B—C10B—C11B	110.0 (4)	C13D—C12D—H12G	109.7
C8B—C10B—C11B	113.2 (4)	C11D—C12D—H12G	109.7
N2B—C10B—H10C	108.0	C13D—C12D—H12H	109.7
C8B—C10B—H10C	108.0	C11D—C12D—H12H	109.7
C11B—C10B—H10C	108.0	H12G—C12D—H12H	108.2
C12B—C11B—C10B	113.0 (5)	C12D—C13D—C14D	110.7 (5)
C12B—C11B—H11C	109.0	C12D—C13D—H13G	109.5
C10B—C11B—H11C	109.0	C14D—C13D—H13G	109.5
C12B—C11B—H11D	109.0	C12D—C13D—H13H	109.5
C10B—C11B—H11D	109.0	C14D—C13D—H13H	109.5
H11C—C11B—H11D	107.8	H13G—C13D—H13H	108.1
C13B—C12B—C11B	111.3 (5)	N2D—C14D—C13D	111.0 (4)
C13B—C12B—H12C	109.4	N2D—C14D—H14G	109.4
C11B—C12B—H12C	109.4	C13D—C14D—H14G	109.4
C13B—C12B—H12D	109.4	N2D—C14D—H14H	109.4
C11B—C12B—H12D	109.4	C13D—C14D—H14H	109.4
H12C—C12B—H12D	108.0	H14G—C14D—H14H	108.0
C12B—C13B—C14B	110.2 (5)	N2D—C15D—C6D	111.7 (4)
C12B—C13B—H13C	109.6	N2D—C15D—H15G	109.3
C14B—C13B—H13C	109.6	C6D—C15D—H15G	109.3
C12B—C13B—H13D	109.6	N2D—C15D—H15H	109.3
C14B—C13B—H13D	109.6	C6D—C15D—H15H	109.3
H13C—C13B—H13D	108.1	H15G—C15D—H15H	107.9
C13B—C14B—N2B	111.8 (4)	O13—Cl1—O14	112.3 (5)
C13B—C14B—H14C	109.3	O13—Cl1—O11	112.0 (5)
N2B—C14B—H14C	109.3	O14—Cl1—O11	111.1 (6)
C13B—C14B—H14D	109.3	O13—Cl1—O12	110.8 (6)
N2B—C14B—H14D	109.3	O14—Cl1—O12	106.1 (7)
H14C—C14B—H14D	107.9	O11—Cl1—O12	104.1 (7)
N2B—C15B—C6B	112.2 (4)	O23—Cl2—O21	100.5 (13)
N2B—C15B—H15C	109.2	O23—Cl2—O22	109.0 (11)
C6B—C15B—H15C	109.2	O21—Cl2—O22	108.4 (11)
N2B—C15B—H15D	109.2	O23—Cl2—O24	112.3 (6)
C6B—C15B—H15D	109.2	O21—Cl2—O24	112.1 (8)
H15C—C15B—H15D	107.9	O22—Cl2—O24	113.6 (5)
C5C—N1C—C1C	122.0 (4)	O31—Cl3—O32	107.0 (7)
C5C—N1C—C9C	122.7 (4)	O31—Cl3—O33	112.1 (5)
C1C—N1C—C9C	115.2 (4)	O32—Cl3—O33	110.5 (6)
C15C—N2C—C14C	112.6 (4)	O31—Cl3—O34	110.8 (7)
C15C—N2C—C10C	113.5 (4)	O32—Cl3—O34	105.4 (8)
C14C—N2C—C10C	111.6 (4)	O33—Cl3—O34	110.8 (4)
C15C—N2C—H2EN	105 (6)	O42—Cl4—O43	113.4 (5)
C14C—N2C—H2EN	111 (6)	O42—Cl4—O41	102.8 (8)
C10C—N2C—H2EN	103 (6)	O43—Cl4—O41	105.1 (6)
O1C—C1C—N1C	119.0 (4)	O42—Cl4—O44	115.1 (6)
O1C—C1C—C2C	124.9 (4)	O43—Cl4—O44	113.9 (3)
N1C—C1C—C2C	116.1 (4)	O41—Cl4—O44	105.0 (6)

### Hydrogen-bond geometry (Å, º)

**Table:**

*D*—H···*A*	*D*—H	H···*A*	*D*···*A*	*D*—H···*A*
N2*A*—H2*AN*···O1*B*	1.03 (5)	1.91 (6)	2.741 (6)	136 (5)
N2*B*—H2*CN*···O1*A*	0.77 (7)	2.00 (6)	2.742 (5)	163 (6)
N2*C*—H2*EN*···O1*D*^i^	0.90 (9)	2.00 (9)	2.735 (6)	138 (8)
N2*D*—H2*GN*···O1*C*^ii^	1.05 (5)	1.74 (5)	2.754 (5)	159 (5)

Symmetry codes: (i) *x*+1, *y*, *z*; (ii) *x*−1, *y*, *z*.
