# Supplementary figures and images for: Crystal structure of anagyrine perchlorate
Source: Acta Crystallogr E Crystallogr Commun. 2015 Apr 25;71(Pt 5):o343–4. doi: 10.1107/S2056989015007781 (PMC4420039; doi:10.1107/S2056989015007781)

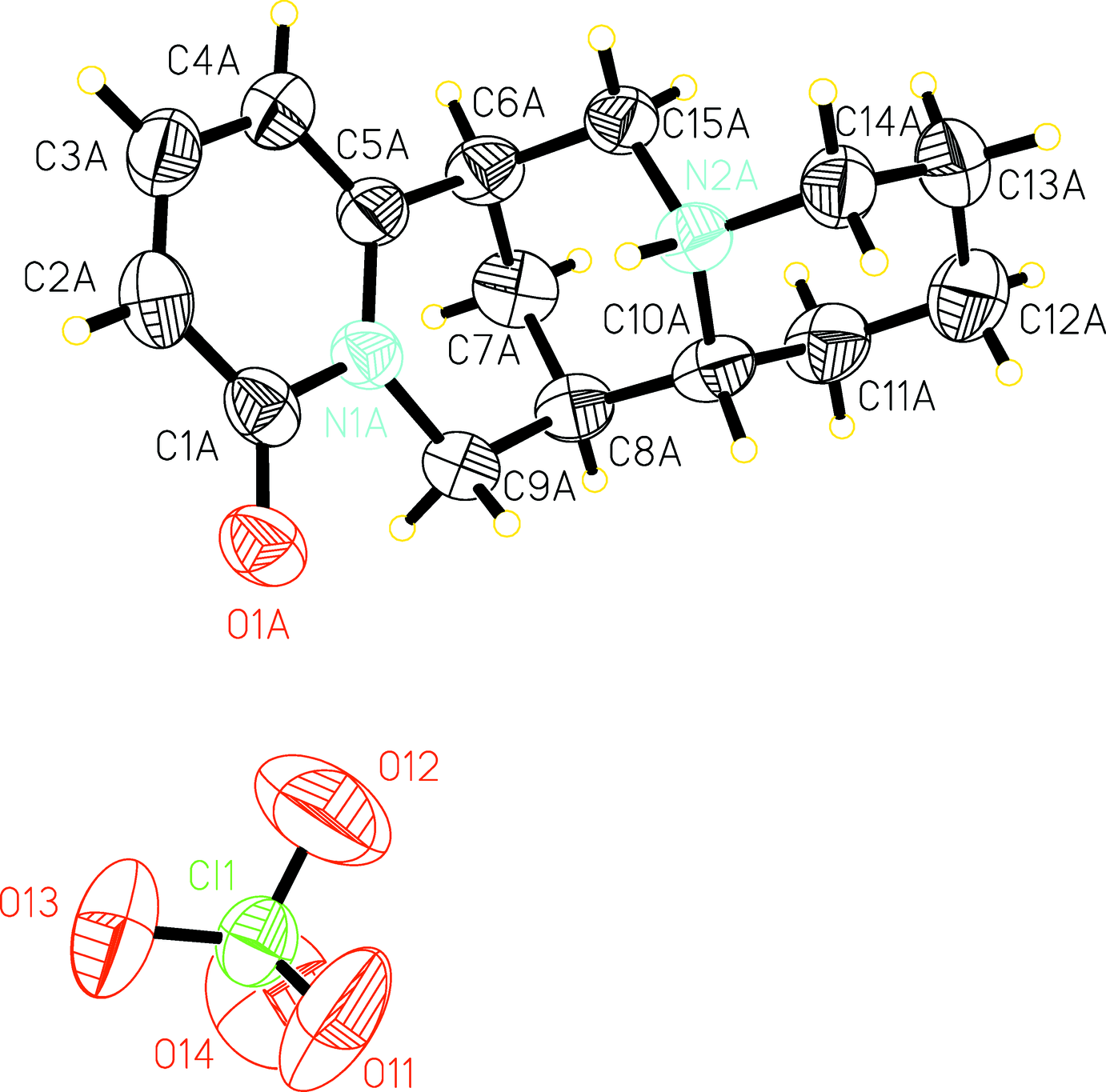

Supplement: Supplementary file 3 [file e-71-0o343-fig1.tif]

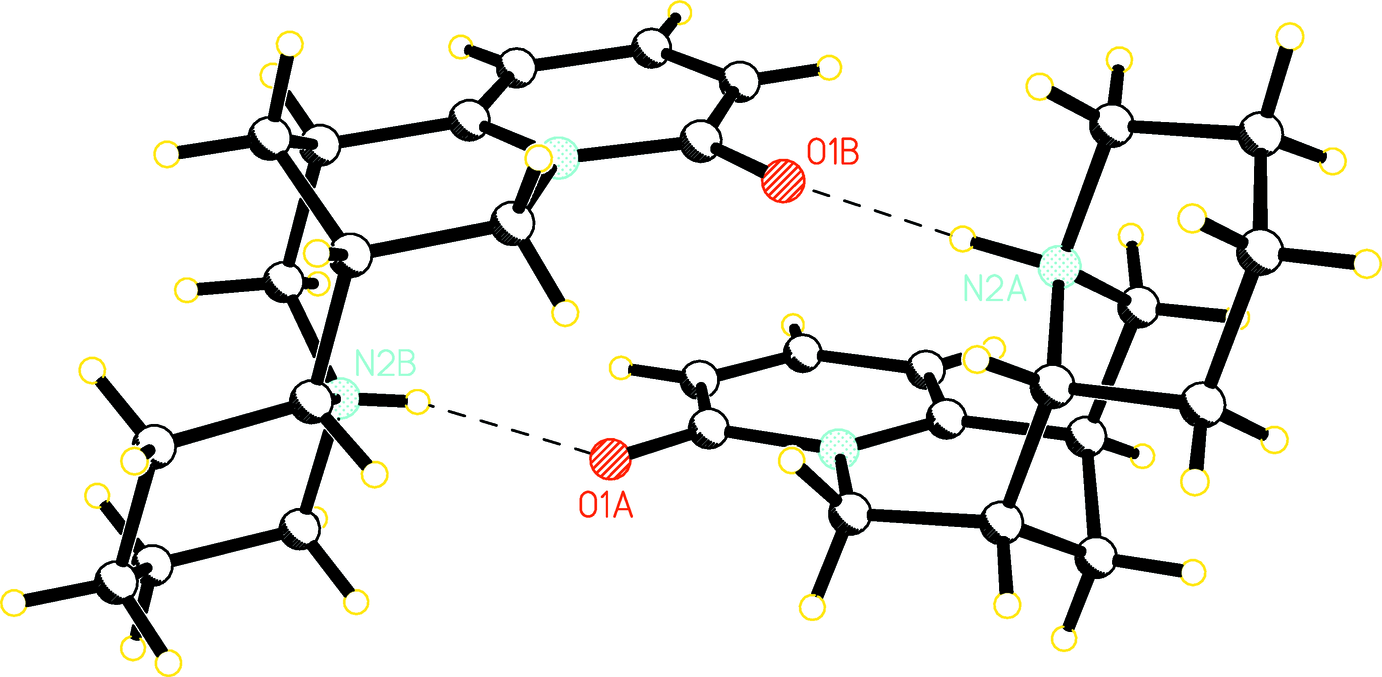

Supplement: Supplementary file 4 [file e-71-0o343-fig2.tif]
